# Predicting Final Extent of Ischemic Infarction Using Artificial Neural Network Analysis of Multi-Parametric MRI in Patients with Stroke

**DOI:** 10.1371/journal.pone.0022626

**Published:** 2011-08-10

**Authors:** Hassan Bagher-Ebadian, Kourosh Jafari-Khouzani, Panayiotis D. Mitsias, Mei Lu, Hamid Soltanian-Zadeh, Michael Chopp, James R. Ewing

**Affiliations:** 1 Department of Neurology, Henry Ford Hospital, Detroit, Michigan, United States of America; 2 Department of Physics, Oakland University, Rochester, Michigan, United States of America; 3 Department of Diagnostic Radiology, Henry Ford Hospital, Detroit, Michigan, United States of America; 4 Department of Biostatistics and Research Epidemiology, Henry Ford Hospital, Detroit, Michigan, United States of America; 5 Control and Intelligent Processing Center of Excellence (CIPCE), School of Electrical and Computer Engineering, University of Tehran, Tehran, Iran; 6 Department of Physiology, Wayne State University, Detroit, Michigan, United States of America; 7 Department of Neurology, Wayne State University, Detroit, Michigan, United States of America; Beijing Normal University, China

## Abstract

In hemispheric ischemic stroke, the final size of the ischemic lesion is the most important correlate of clinical functional outcome. Using a set of acute-phase MR images (Diffusion-weighted - DWI, T_1_-weighted – T1WI, T_2_-weighted-T2WI, and proton density weighted - PDWI) for inputs, and the chronic T2WI at 3 months as an outcome measure, an Artificial Neural Network (ANN) was trained to predict the 3-month outcome in the form of a voxel-by-voxel forecast of the chronic T2WI. The ANN was trained and tested using 12 subjects (with 83 slices and 140218 voxels) using a leave-one-out cross-validation method with calculation of the Area Under the Receiver Operator Characteristic Curve (AUROC) for training, testing and optimization of the ANN. After training and optimization, the ANN produced maps of predicted outcome that were well correlated (*r* = 0.80, *p*<0.0001) with the T2WI at 3 months for all 12 patients. This result implies that the trained ANN can provide an estimate of 3-month ischemic lesion on T2WI in a stable and accurate manner (AUROC = 0.89).

## Introduction

In hemispheric ischemic stroke which is caused by blockage of a blood vessel, the clinical outcome is strongly related to the final size of the ischemic infarct [1], [2], [3]; generally speaking the more extensive the damage to the brain parenchyma, the worse the patient's clinical outcome is likely to be, although this is of course also dependent on location. As a measure of tissue recovery, and strongly related to patient outcome, MRI T2-weighted imaging at 3 months post-ictus is generally accepted as the gold-standard [4]. Consequently, during the acute phase of ischemic stroke, a fast and reliable identification of the ischemic lesion, its extent, and a prediction as to its fate, might aid clinical decision-making and help to maximize benefit and minimize side effects of a therapeutic intervention [5], [6]. Many studies, by correlating with follow-up imaging or neurological status, have shown the potential for diffusion weighted imaging (DWI), perfusion weighted imaging (PWI), and/or T_2_-weighted (T2WI) magnetic resonance imaging (MRI) used together for staging stroke outcome [3], [7], [8]. Most of these analyses were performed using as an outcome metric the volume of a region-of-interest (ROI) defined by the difference between an acute data set and the outcome image [9], [10], [11]. For instance, it has been shown that an unsupervised clustering technique such as ISODATA (Iterative Self-Organizing Data Analysis) can utilize combined MRI data sets from the acute phase [2], [12] and the subacute phase [5] post-stroke to predict final infarct volume and thus produce a time-independent surrogate MRI outcome predictor [13], [14], [15], [16], [17]. However ISODATA has some significant drawbacks.

Maps produced in ISODATA are sharply delineated, and not approximately continuous as in the outcome measure, the T2WI [18]. Furthermore, to a large extent, ISODATA mapping and standardization does not produce an easily visualized association between the map and the outcome measure. Additional problems in the ISODATA technique include: ISODATA's instability in the presence of image artifacts and noise, its sensitivity to initial conditions, uncertainty as to the underlying variances of the clusters, and its dependence on the assumption of normality for the distribution of clustered data [19]. Thus, ISODATA and related approaches, while useful, do not provide a voxel-by-voxel judgment as to the potential for eventual infarction; finding a continuous predictor for infarction remains an open problem [20], [21].

Statistical approaches such as Probability-of-Infarct Profile (PIP) and Generalized Linear Model (GLM) algorithms constructed based on ADC and CBF maps (in animal models of acute stroke), or combining DWI and PWI MRI (in patients), have shown good sensitivity and specificity in predicting the outcome in brain parenchyma after stroke [22], [23]. The success of these methods suggests that probabilistic voxel-based approaches may have a significant predictive ability, and therefore may be useful in predicting response to therapies administered in the immediate aftermath of a catastrophic cerebral ischemic event.

Combining GLM and Spatial Auto Regressive (SAR) models may help improve the predictive ability as to the fate of infracted parenchyma [24], [25]. However, this process is not fully automated, since it requires the acute stroke area to be identified by a neurologist. In terms of establishing its coefficients, the GLM-SAR methods demand a large sample size to generate a reliable estimate of model parameters.

In a recent study [26], the final infarct volume in patients treated with IV rt-PA (recombinant Tissue Plasminogen Activator) was predicted by an artificial neural network (ANN) model that combined both clinical and imaging variables [National Institutes of Health Stroke Scale (NIHSS) and DWI images]. Although this study demonstrated that an ANN was capable of predicting tissue fate using acute information, there was no voxel-by-voxel prediction of outcome; rather this study focused on prediction of chronic lesion size regardless of its pattern, location and distribution.

This paper presents an ANN model that incorporates an easily obtained data set, and one that is not strongly technique-dependent, to predict the stroke outcome with a power of prediction that approaches the methods discussed above. While the paper does not explicitly address the matter of the minimal MRI data set required for prediction of tissue outcome, it is notable that a reasonably strong predictive power was achieved in a data set that did not include an acute PWI set. The ANN technique thus provides a very fast (essentially real-time), approximately continuous, and intuitive mapping of the predicted outcome, while preserving the time-independent and multi-parametric strengths of clustering approaches.

## Materials and Methods

### Ethics Statement

This research has been approved by the Henry Ford Health System Institutional Review Board. We obtained written informed consent from all participants in the study.

### Hypothesis

We hypothesized that, given a T2WI at the chronic stage of stroke (3 months post-stroke), an ANN might be trained to directly predict the size and pattern of the tissue recovery from the information available in acute phase MR images.

### Patients and MRI studies

Twelve patients (with a total of 83 slices) with acute neurological deficit consistent with ischemic stroke, and MRI studies within 24 hours of onset (defined as the last time the patient was known to be without neurological deficit), were selected. The severity of neurological deficit was assessed at the time of the MRI study using the NIHSS score. MRI studies were performed at the acute time point (<24 h post-ictus), and at outcome (3 months post-ictus). Patients with cerebral hemorrhage, a history of prior stroke or other neurological disorders hampering interpretation of the neurological outcome were excluded. The study was approved by the Institutional Review Board.

An acute-phase MR image set consisting of a T_1_-weighted (T1WI), T2WI, DWI and proton density (PDWI) was selected as the input set to the ANN. The co-registered three month T2WI, considered to be the gold standard for final infarct size (and estimation of the tissue recovery), was employed as the training set.

MRI studies were acquired on a 1.5-Tesla GE Signa MR scanner with echo-planar capability (GE, Milwaukee, WI). Each MRI study consisted of axial multispin echo T2WI, and DWI with slice thickness 6 mm. The field of view (FOV) was 240×240 mm. For T1WI and T2WI, the matrix was 256×192 and for DWI 128×128. Additional parameters for each study were: (a) T1WI: TR/TE = 600/14 ms; (b) T2WI: TR/TE = 2,800/30, 60, 90, 120 ms; (c) axial DWI was performed using an echo-planar sequence, TR/TE = 10,000/101 ms, b-values = 1,000, 600, 300, 0 s/mm^2^, sequential application of three separate diffusion-sensitizing gradients in perpendicular directions, 1 NEX.

For each patient, four image sets (T1WI, T2WI, DWI and PDWI) at the acute time point were selected to provide input features to the ANN. All acute- and chronic phase images were registered to the acute T2WI using Eigentool software [27]. To reduce mis-registration effects, images were smoothed using a unity filter with a 3×3 window, and the smoothed images were then normalized to their mean value, thus creating a feature set insensitive to the MR system gain.

ROIs were defined by thresholding the 3-month T2WI to outline the region of infarction. This region was reflected around the midline and adjusted so that the resultant region selected a visually normal contralateral area of tissue, so as to approximately balance normal and infarcted tissue. Prevalence which refers to the ratio of number of samples from the lesion area divided by the total number of samples from the lesion and normal areas was about 57%. The selected infarcted and normal regions were used together as inputs to the ANN.

### Input and Training Set for the ANN

**Figure 1:**
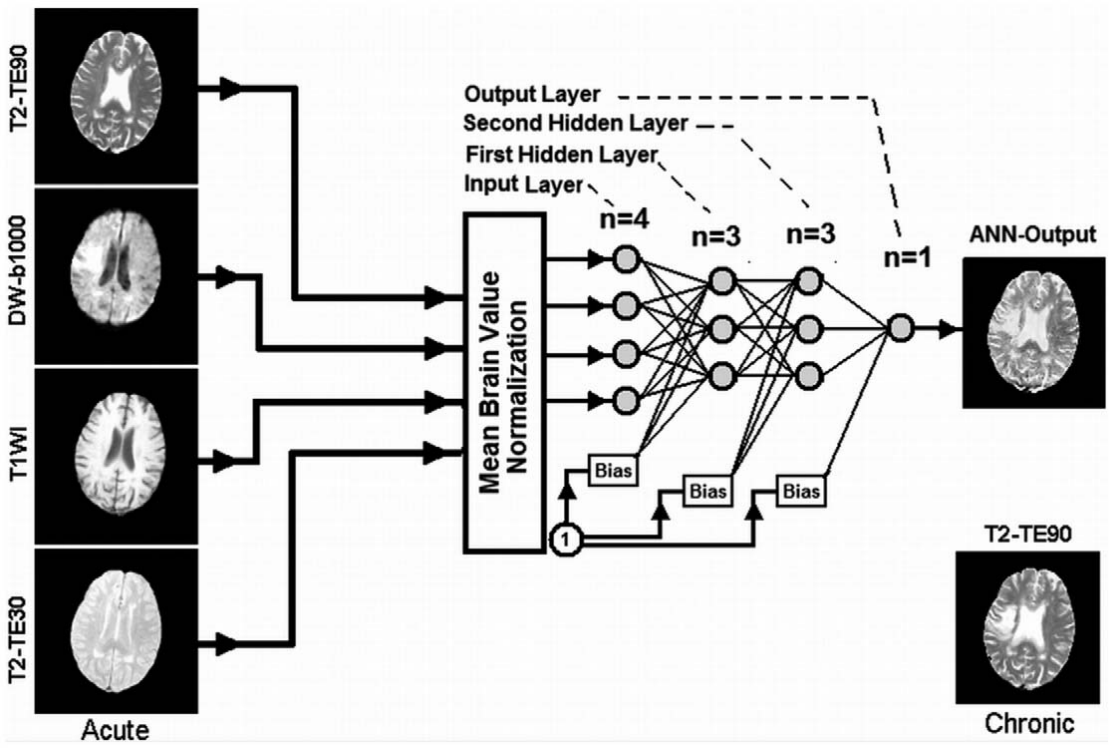
ANN diagram for phase of training and prediction. As shown in this figure, 4 MR image modalities are input to the ANN to predict T2WI at three month. Note that the MR modalities are normalized to their brain mean values before feeding to the ANN.

Using the normalized data from the selected ROIs (83 slices, and 140218 voxels, representing total number of the training samples for all subjects) drawn by the study neurologist (PDM), a feature set was generated from the four selected acute-phase images and presented to a feed-forward multilayer perceptron (MLP) with back propagation training algorithm as an input vector. In this type of ANN, as shown in Figure 1, the nodes are organized in the input layer, hidden layers, and the output layer. Nodes are interconnected by weights in such a way that information propagates from one layer to the next through a sigmoid (bipolar) activation function. Learning rate and momentum factors control the internode weight adjustment during the training. A back propagation learning strategy [28], [29], [30] was employed for training the ANN in a supervised mode. In this strategy, a trial set of weights (the weight vectors, one vector for each layer of the ANN) is proposed. The input vectors (a set of 4 voxels extracted from 4 normalized modalities such as DWI, T1WI, T2WI and PDWI) are presented to the ANN, and the output result compared to the class identifier (in our case, this was the co-registered T2WI image at three month study). The weight vectors are then adjusted to minimize some measure of error, i.e., the Mean Square Error (MSE) between the output of the ANN and the normalized T2WI at three months (training set). This procedure is performed iteratively across the entire data set.

Batch processing was used to improve the convergence rate and the stability of training. The weight changes obtained from each training case were accumulated, and the weights updated after the entire set of training cases was evaluated. Batch processing improves stability, but with a tradeoff in the convergence rate [30], [31]. A leave-one-out cross-validation method was employed for training, testing, and network optimization [31], [32] where the one in the leave-one-out was all data, i.e., all multiple voxel image sets in an entire patient study.

The leave-one-out validation method is a member of the set of cross validation methods, where all data are used for fitting (but not at the same time, of course). Its prediction is based on a large data set, which leads to small prediction errors, and is superior for small data sets, compared to data split-sample validation [33].

### ANN Optimization and Calculation of the ANN Generalization Error

To generalize the ANN, *i.e*., to allow its application to a wide range of inputs, we needed to avoid both under-fitting of the training data (which generates a high variance in the output estimate) and over-fitting of the training data (which produces biased outputs). There are a number of strategies for assuring generalization:

1. Optimize the number of free parameters (independent connection weights) in the model (*e.g*., the number of neurons in each layer and the number of layers).
2. Stop the gradient descent training at an appropriate point.
3. Add noise to the training patterns to smooth out the data points.

Strategy number 3 is employed in cases where local minima “trap” the ANN optimization process. Since no trapping was observed, strategy number 3 was not employed in this study. To employ strategies number 1 and 2, we must estimate from our training data what the generalization error is likely be. A leave-one-out method was employed for training, testing, and network optimization [30], [31], [32], [34]. To characterize the generalization error, we trained and validated the ANN by the leave-one-out method, which is a special case of the K-Fold Cross Validation (KFCV) method, using the area under the receiver operating curve (AUROC) as a cost function [31], [32], [35], [36].

In this study, each fold consisted of one full case (patient), thus the K folding cross-validation (KFCV) method was that of leave-one-out. The leave-one-out method is one of the most powerful versions of the KFCV method; it has been shown that the Correct Classification Fraction (CCF) of the leave-one-out method is an important statistical estimator of the performance of a learning algorithm [37]. The performance of the leave-one-out method is frequently used for estimation of the generalization error [38], [39]. In KFCV, the training data are divided into *K* distinct subsets (*K* = 12, one case in each fold); the network is then trained using *K*-1 subsets, and tested on the remaining subset (the left out case). The process of training and testing is then repeated for each of the *K* possible choices of the subset omitted from the training. The average Correct Classification Fraction (CCF) versus different epochs (epoch refers to each trial and error for decreasing the MSE of the ANN in the batch processing mode) for the *K* omitted subsets was plotted and the epoch corresponding to 10% of its plateau was taken to be the stopping epoch. The MSE of the ANN for all *K* subsets at different stopping epochs was calculated and its average value was taken as the ‘stopping error’ for the optimal ANN.

This procedure has the advantage that it allows us to use a high proportion of the available training data, a fraction (1–1/*K*), for training, while making use of all the data points in estimating the generalization error or agreement. The cost is that the process can be lengthy, since we need to train and evaluate the network *K* times. Typically, *K*≈10 is considered reasonable [32], [36]. In this study, *K* was set to 12 for 12 patients (one case in each fold) and the ANN had a single output, to predict a T2WI at the chronic time point.

**Figure 2:**
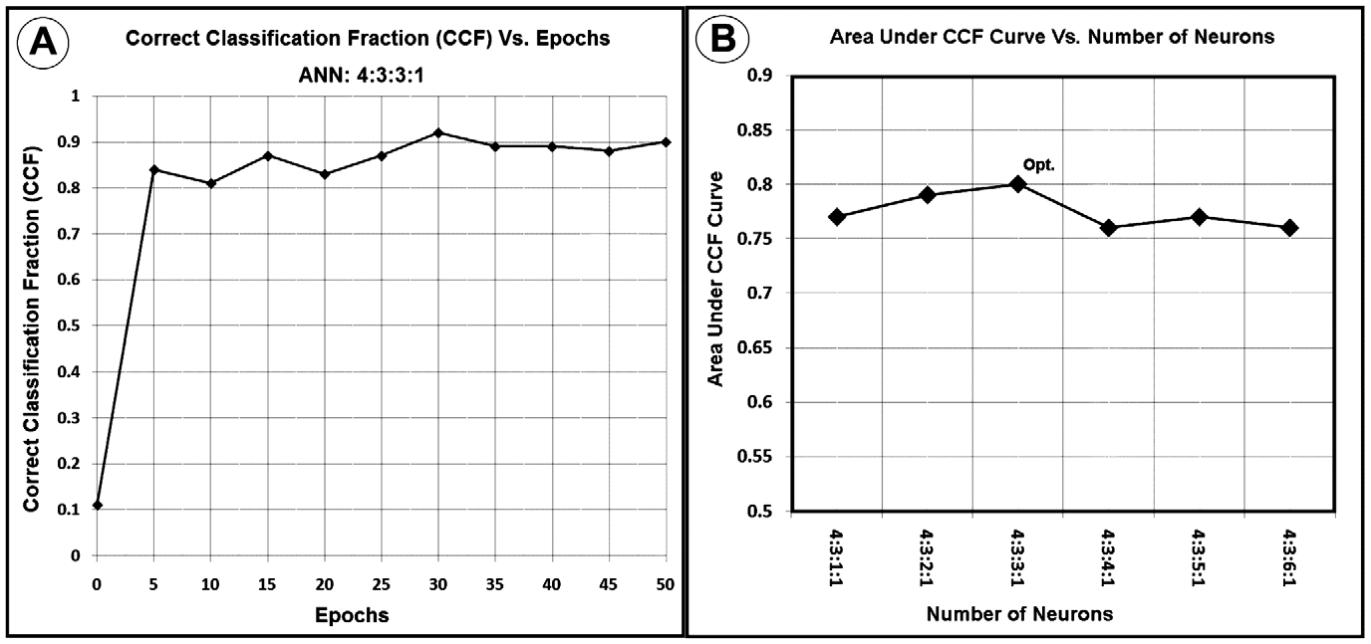
A Correct Classification Fraction versus epochs for the optimal ANN (4∶3∶3∶1) using 83 slices of twelve patients with 12 folds. Note the termination epoch is around 35, which is corresponding to 10% of CCF curve plateau. 2-B: Area Under Correct Classification Fraction (AUCCF) curve versus number of neurons in the second hidden layer. As shown in this figure, AUCCF is maximum for three neurons in the second hidden layer.

To measure how accurately this ANN matched the whole input dataset (each set of MR modalities for all voxels) with the entire outcome set (each voxel in all 3-month T2WI studies), as shown in Figure 2-A, the ANN's CCF which is the ratio of the accumulated number of voxels successfully classified by the ANN, divided by the number of all voxels shown to the ANN at specific epoch was generated at different levels of epochs during the leave-one-out validation procedure.

True-Positive-Fraction (TPF), sensitivity, is the fraction of voxels that actually are lesion and are correctly predicted as lesion. True-Negative-Fraction (TNF), specificity, is the fraction of voxels that actually are normal and are correctly predicted as normal. False-Positive-Fraction (FPF) is equal to (1-specificity) and is considered as the confidence level (CL). Thus at each confidence level (1-specificity), the AUROC is proportional to the TPF which is proportional to the Correct Classification Fraction Each [36], [40]. The Area Under the Correct Classification Fraction (AUCCF), which is proportional to the AUROC (*A_z_*) value, was used as an index to compare the ANN's performance, to determine the optimal architecture of the ANN, and to find the ANN termination error [36], [40]. True-Positive-Fraction (TPF), sensitivity, is the fraction of voxels that actually are lesion and are correctly predicted as lesion. KFCV (leave-one-out) set was trained until its error was below a defined termination error (i.e., the point at which the training procedure was stopped). The termination error was set by determining the error at the 10% point of the CCF's plateau.

The number of hidden layer nodes may affect the performance of the ANN classifier [32], [35]. Since the ANN could not be trained by less than 3 neurons in its first hidden layer, layer and node optimization were done by maximizing the AUCCF value for its second hidden layer as a function of the number of nodes.

Internally, in the ANN, the activation function of the ANN neurons is a sigmoid function that is most easily programmed to work in a polar mode between −1 and 1; the output of the single output neuron is a continuous function with a mean of 1 (range 0–3), since the training set T2WIs were normalized to their mean value (mean value of the brain). To test, optimize and validate the ANN using the CCF, the minimum acceptable interval for the T2WI signal intensity change was calculated and then the values of the ANN output and the gold standard maps of training (T2WI) both were broken down into discrete fragments using a small window (0.05). In order to present an easily understood comparison, the response of the trained ANN was compared to the chronic T2WI (gold standard) by calculating a correlation coefficient using all of the voxel comparisons (with 0.05 windows) available and ROC curve of the optimal ANN.

To study the early prediction of ANN MRI at acute phase to the chronic ischemic infarction at 3 months after stroke, the linear regression model was used based on voxel data (*n* = 140218), adjusted for cluster (patient, *N* = 12) using PROC SURVEYREG in SAS [41]. The analysis tested significant correlation of the acute ANN MRI to 3-month infraction; followed by calculation of the correlation coefficient, *r* for predictive ability.

## Results

The demographic and clinical characteristics of the patients included in the study are summarized in table 1. The mean age was 56.25±11.56 years. The ischemic stroke subtype was: cardioembolsim (n = 2), large vessel atherosclerosis (n = 3), small vessel disease (n = 3), cryptogenic (n = 4). The time interval from symptom onset to acute-phase MRI study was 11.32±4.36 hours, and from symptom onset to outcome MRI study was 95.75±22.54 days.

**Table 1:** Demographic table.

Pt	Age/Gender	Stroke Location	Ischemic Stroke Subtype	Onset-Acute MRI (hours)	Onset-Outcome MRI (days)
1	42/M	Left frontal	Large vessel atherosclerosis	11.8	90
2	68/M	Left basal ganglia-internal capsule	Small vessel disease	9.3	95
3	42/M	Left corona radiata - internal capsule	Small vessel disease	10.6	103
4	59/M	Left corona radiata-thalamus	Cryptogenic	13.4	93
5	45/M	Right frontal-parietal-temporal	Cardioembolism	8.2	93
6	59/F	Left internal capsule-thalamus	Small vessel disease	8.2	104
7	68/M	Right frontal-parietal	Large vessel atherosclerosis	10.9	82
8	45/M	Right parietal	Cryptogenic	10.1	102
9	64/F	Left frontal-parietal	Cryptogenic	18.4	130
10	71/F	Right frontal-parietal	Large vessel atherosclerosis	5.4	39
11	67/F	Right frontal-parietal	Cardioembolism	20.7	124
12	45/M	Right occipital-temporal	Cryptogenic	8.9	94

Training and validation objectives of the ANN were accomplished in the KFCV (leave-one-out) method. As in Figure 1, a set of 4 feature vectors extracted from DWI, T1WI, T2WI and PDWI were presented to the ANN and the performance of the ANN with respect to its number of second hidden layer nodes was examined by considering the AUCCF value at a Leave-One-Out termination error (MSE) of (MSE = 0.06∼35 epochs).

The ANN was trained and tested for a set of normal and lesion ROIs. For a statistically reliable comparison, and to increase the ANN's accuracy for lesion detection, map sampling was done at a prevalence of about 57% by choosing regions of interest (ROIs) from normal tissue with about the same area of the lesion. The optimal ANN ([4+1 bias] ∶ [3+1 bias] ∶ [3+1 bias], [1]) was found by maximization of the AUCCF value for one hidden layer. In Figure 2-A, a plot of the CCF curve (for the optimal ANN; 4∶3∶3∶1) and in Figure 2-B the AUCCF vs. the number of neurons in the hidden layer for a stopping error of 0.06, learning rate of 0.01 and momentum of 0, are shown. As Figure 2-B demonstrates, the maximum value of the AUCCF (∼0.80) gives the optimal number of neurons (three neurons plus one bias) in the second hidden layer.

**Figure 3:**
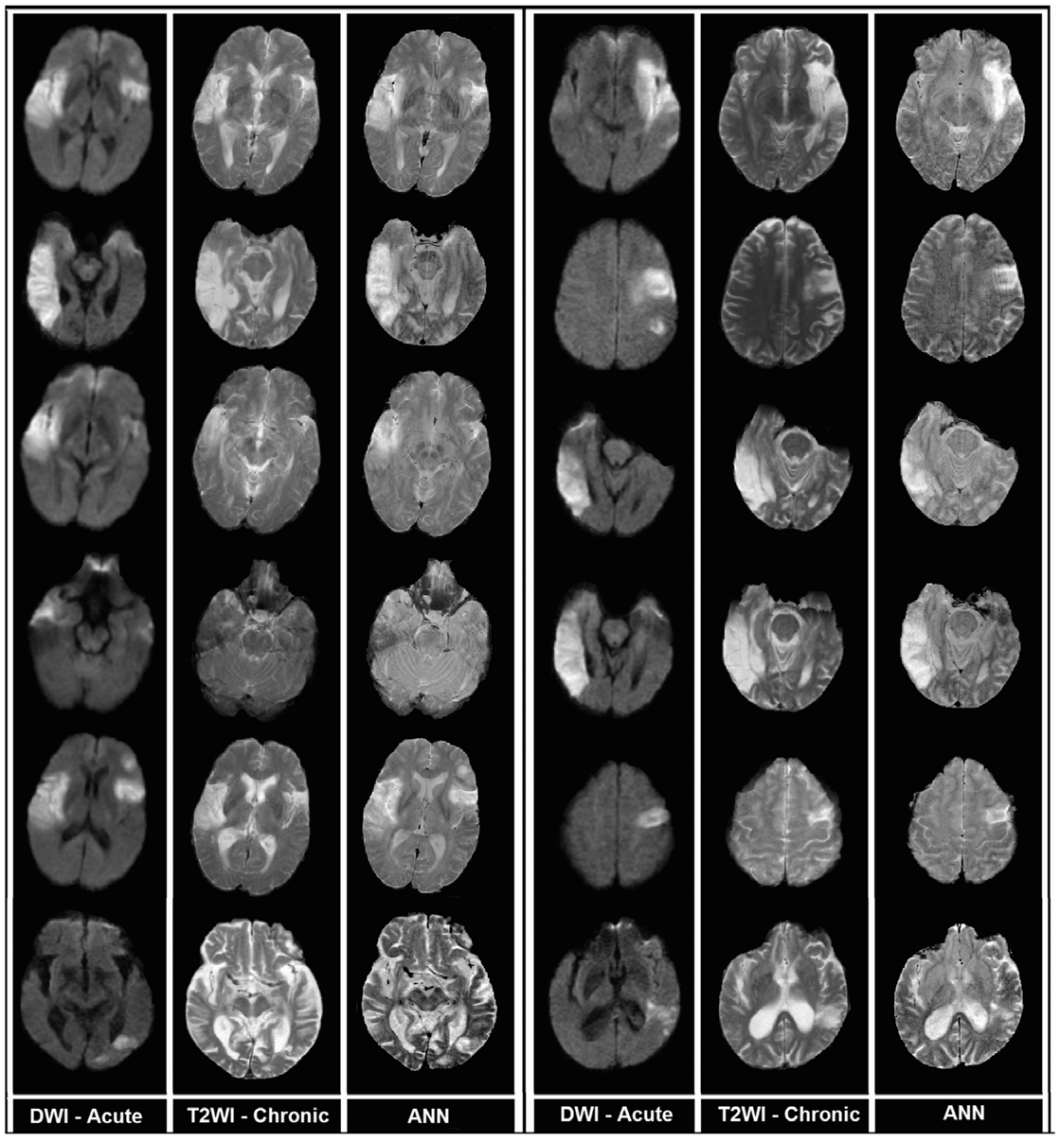
Examples of ANN-produced images in 12 exemplary MRI slices from 9 patients. Left column: DWI in acute study. Middle: outcome T2WI. Right: ANN predicted outcome from acute image set. The last slices in both columns present the least reliable prediction of the ANN.

Using all 12 patients (83 slices, and 140218 voxels), the trained and optimal ANN ([4+1 bias] ∶ [3+1 bias] ∶ [3+1 bias],[1]) generated maps that were well correlated (*r* = 0.80, *p*<0.0001) with the chronic T2WI. Figure 3 contains twelve exemplary (from 9 different patients) whole-brain voxel-wise results, generated by the trained ANN, showing the acute DWI study (left-hand column), the chronic T2WI (middle column), and the ANN outcome predicted by the acute image set (right-hand column).

As Figure 3 demonstrates, the whole-brain voxel-wise predictions generated in seconds by the trained and optimal ANN, are visually similar to their corresponding chronic T2WI; the pattern of the lesion predicted by the ANN are well matched with their three-month lesion in both training and test examples. Note that the continuity of the ANN output provides a more graded prediction regarding the outcome tissue viability compared to other statistical techniques such as ISODATA. Although the ANN predictions in most of the cases shown in Figure 3 are highly correlated with their corresponding T2WI, the ANN predictions in the last two cases of both columns are not strongly in agreement with their corresponding T2WI. The possible sources of these errors are discussed below.

**Figure 4:**
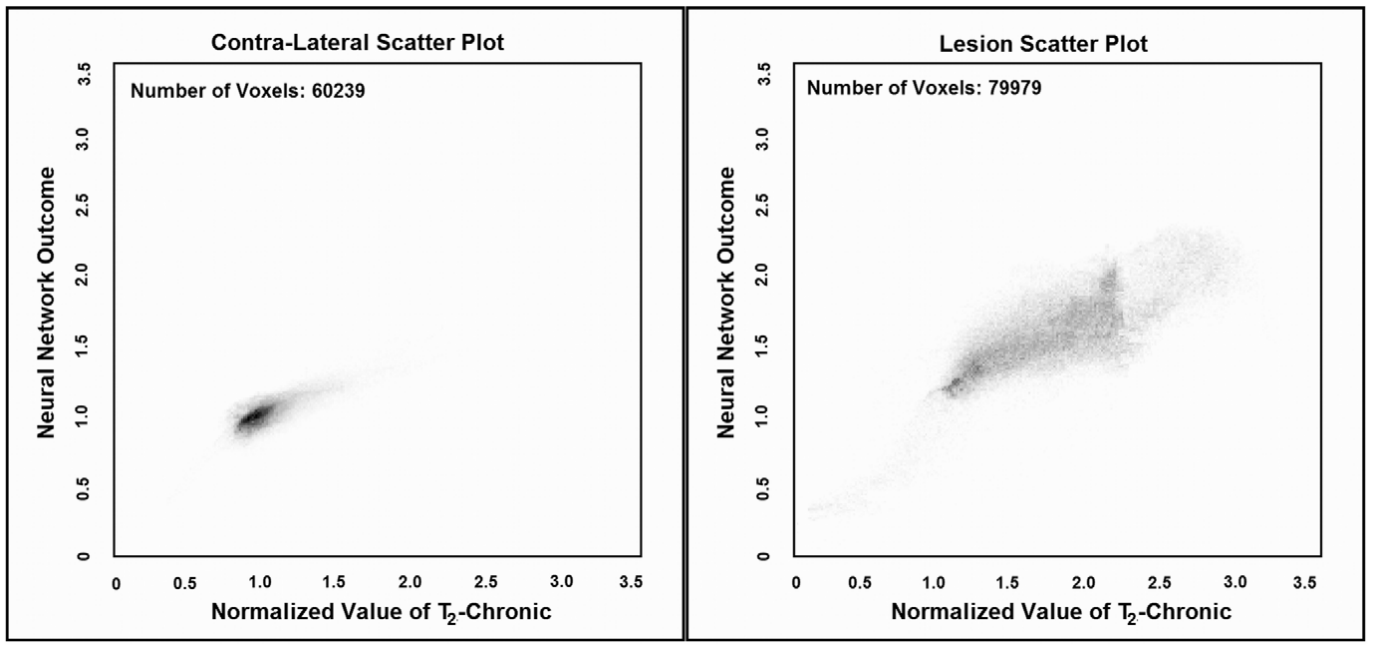
Scatter plots of the optimized ANN (4∶3∶3∶1) for predicting lesion and normal areas of 83 slices in 12 patients compare to their T2WI at chronic (gold standard). As shown in these figures, the ANN prediction is highly correlated (Total voxels: 140218, r = 0.80, p <0.0001) with the T2WI at three month.

Figure 4 presents scatter plots of the ANN response versus T2WI image at three months for lesion and control areas for all 12 patients. Note that in the normal scatter plot, there is a cluster which is formed below 1.0 with a less scattered pattern. However, in the lesion scatter plot, a cluster is formed above 1.0 with more scattering and less density compared to the normal area.

More scattering pattern in the lesion plot represents the capability of the ANN for estimating different levels of lesion with different classes or intensity. The small values observed in the lesion scatter plot are associated with the mis-registration errors and the large values are associated with CSF area since the ANN is forced to classify the CSF and lesion at the same time.

**Figure 5:**
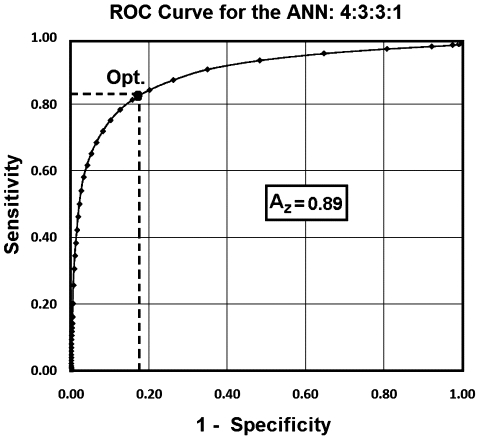
ROC curve (AUROC = 0.89) for the trained ANN (4∶3∶3∶1). As shown in this Figure, the optimal sensitivity and specificity of the ANN are calculated at the half angle and ROC curve intercept point (Point Opt.; Specificity Optimal  = 0.84, Sensitivity Optimal = 0.85) which implies that the ANN can predict lesion and tissue with a sensitivity of 85% and a specificity of the 84%.

As noted, the ANN prediction for all 12 patients was highly correlated (*r* = 0.80, *p*<0.0001) with the T2WI outcome measure at three month. To estimate the overall performance of the trained ANN, the ROC curve of the trained ANN (4∶3∶3∶1) was generated for all training samples and the Area Under ROC (AUROC = 0.89, Average error = 0.11) was considered as the performance of the trained ANN (see Figure 5). As shown in Figure 5, the optimal sensitivity and specificity of the ANN were calculated at the half angle and ROC curve intercept point (Specificity^Optimal^  = 0.84, Sensitivity^Optimal^ = 0.85) which indicates that the ANN can predict lesions with a sensitivity of 85% at a specificity of 84%.

**Figure 6:**
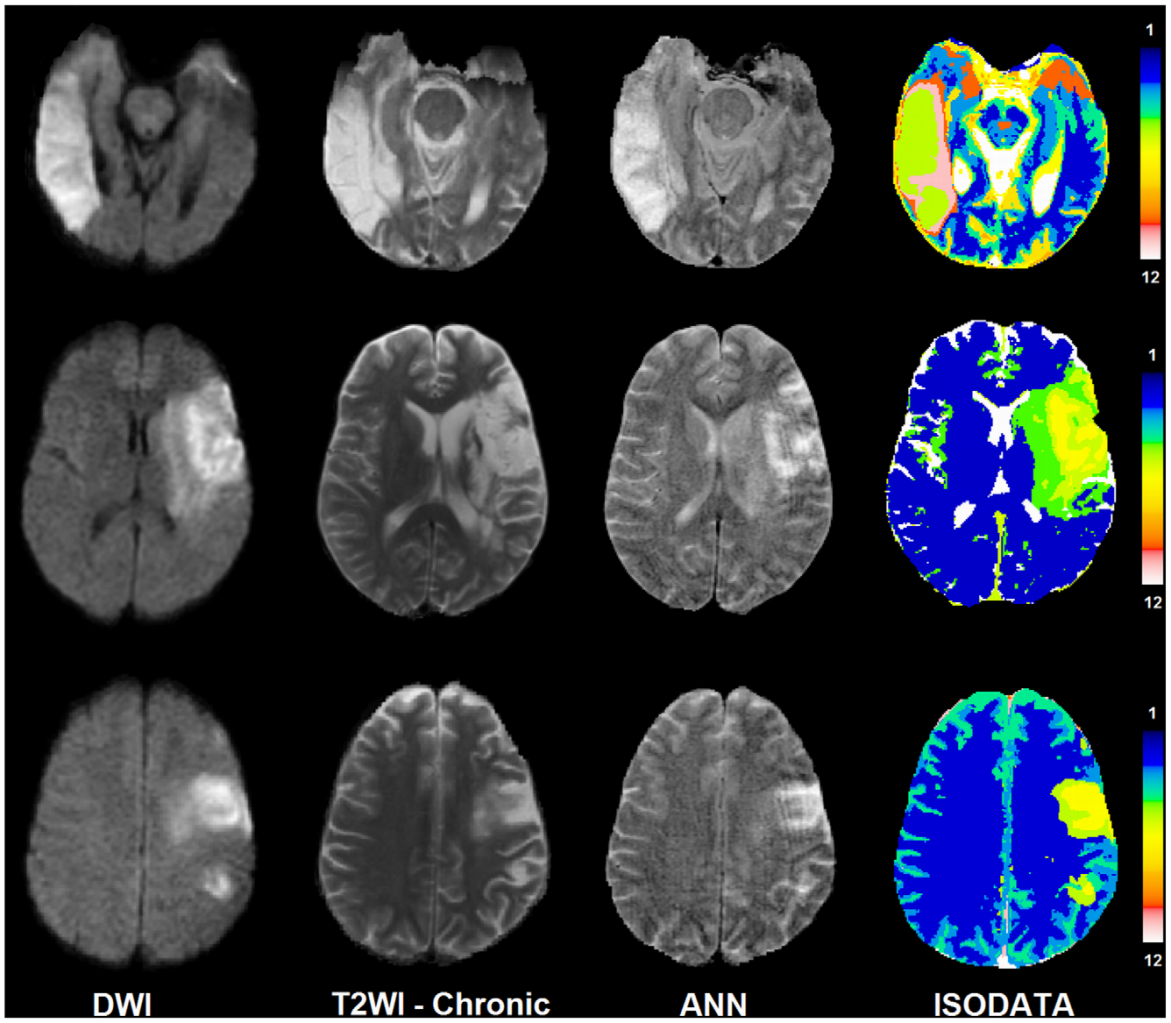
This figure presents DWI, T2WI-Chronic, ANN, and ISODATA maps for 3 different patients. In ISODATA maps the lesions are clustered into 2 to 3 different signatures [from top to bottom: (5, 6, and 8), (2, 3, and 4), (3 and 4)]. Signature number indicates the chance of tissue recovery. The higher the signature values, the lower the chance of recovery.

Figure 6 compares the results of the ISODATA technique with those of the ANN. As shown in this figure the lesion areas in the ISODATA maps are clustered to different signatures (1–12). Signatures range between one (corresponding to normal tissue) to twelve (corresponding to CSF or cavitated tissue). While the ANN demonstrates a continuous map for the status of the tissue at risk in the lesion area, the ISODATA shows just a few indices related to tissue recovery after three months. Both techniques are in agreement in the CSF area or dead tissue.

## Discussion

Using a relatively small set of acute-phase MR images, an ANN was trained and tested to directly predict the size and pattern of the eventual tissue damage, as judged by T2WI at the chronic stage of stroke (3 months after stroke onset). Since the predictor produced by the ANN provides a visual estimate of the outcome, such modeling may play an important role in the assessment of therapeutic interventions applied within extended therapeutic windows, currently of great interest in the treatment of stroke.

It must be noted that the training set was small (12 cases) in the number of patients; that is why the leave-one-out method was used for training and evaluation. However, even this small pilot study demonstrates a robust and sensitive predictor of infarct outcome, and because it can be produced almost as quickly as the image sets emerge from the MRI scanner, this approach shows great potential as a tool that may eventually influence clinical practice. Although the ANN in this study was trained and validated with a small sample size, the values of the AUROC (0.89), the optimal specificity and sensitivity (0.84 and 0.85), and the average error (0.11) achieved with the leave-one-out technique supports the hypothesis that the ANN can reasonably predict lesions if it is applied to a new dataset.

Since the ANN uses acute-phase information, any processing errors due to mis-registration, image deformation due to edema created by the lesion between different time points, image quality, partial volume effects, image artifacts, and image uniformity are potential sources of errors that can affect ANN results.

The results of the ANN implies that the trained ANN is a robust discriminator between CSF and lesion areas since the intensities of the CSF (dark) and lesion (bright) areas appear oppositely in DW images but the same in the maps predicted by the ANN. However, this might be a problem for the closely located lesions to the CSF areas which can be easily confused in the interpretation phase by means of a non-expert user (not the ANN).

The GLM-SAR study [24] [25]showed that, compared to algorithms using DWI or PWI separately, algorithms combining acute DWI and PWI were superior in predicting the extent of final tissue infarction. GLM models have the drawback of assuming class boundaries to be hyperplanes in feature space. This fundamentally linear model of response may not suffice to account for the complex dependency of infarct risk upon the combined metabolic and structural derangement reflected in the MR perfusion and diffusion indices in ischemic tissue.

Clinical neurological recovery, as measured by the change in NIHSS, can also be used as additional information for training the ANN in acute to chronic time point [26]. The model presented by Luby et al., was generated by a generalized regression neural network (GRNN) trained using such baseline information as sex, age, NIHSS, stroke onset time to MRI scan time, and corresponding values for follow-up: modified Rankin Score (mRS), follow-up NIHSS, baseline lesion volume on diffusion weighted MRI, baseline hypoperfusion volume (on MTT map), follow-up reperfusion (reduction of MTT volume by at least 30%), and final infarct volume on FLAIR MRI. The GRNN approach offers the possibility that our study, focused as it was on the estimation of the lesion volume regardless of its pattern and location in the brain, could further benefit from additional clinical and imaging information; it may be possible to form a more clinically relevant prediction of outcome by including the NIHSS score as an input to the ANN, in addition to the feature set currently in use.

Most of multi-parametric analysis techniques such as ISODATA, Fuzzy clustering, and Kohonen's Multi-Parametric Self-Organizing Map (KMP-SOM), employ the theory of information extraction for predicting tissue fate at three months using MR acute information, regardless of chronic information. The chronic information is used just for validation and assessment of these techniques. To date the only method that we know of that has used chronic information for segmentation prediction is the GLM-SAR [24]. As we have noted, there is still a remaining question as to whether a linear model is inherently the correct model for stroke outcome prediction. Unlike the GLM-SAR, the ANN combines acute information with no prior assumption about the linearity of information in its combination.

A fast, robust, and time-independent predictor of stroke outcome raises the possibility of real-time evaluation of evolving stroke in the acute and subacute phase. We will assess the possibility that the ANN predictor of outcome T2WI is essentially time-independent, because the acute/subacute T2WI image, with its increasing contrast with time post-ictus, can independently provide a measure of the post-stroke duration.

This study used untreated patients to train an adaptive ANN for predicting the tissue fate in absence of any drug or treatment. Its performance in treated patient populations remains to be assessed. We speculate that if, the trained ANN uses the MR information of treated patients before and after the course of treatment, differences between the predicted outcome lesions (prior to, and after, treatment) might be a measure of treatment efficacy. Thus, given effective treatments, a real-time predictor of outcome can produce a paradigm shift in the treatment of stroke in all its early stages, and may ameliorate or avoid many of the profoundly destructive sequelae of cerebral infarction.

In the sense that all patients had in common MRI images with both normal and ischemic regions of brain, and it is to be expected that normal brain and ischemic brain have similar MRI presentations across patients, the sample size can be considered to be large (83 slices and 140218 voxels). However, we have to acknowledge that ischemic stroke patients may show a range of MRI presentations, and that the sample of patients chosen, while of manageable size for a proof-of-principle study, needs to be enlarged for further evaluation of the stability of the predictor.
